# Temporal and Depth‐Driven Variability of Pelagic Bacterial Communities in Lake Erie: Biofilm and Plankton Dynamics

**DOI:** 10.1111/1758-2229.70079

**Published:** 2025-03-21

**Authors:** Rylie L. Robinson, Aaron T. Fisk, Sophie Crevecoeur

**Affiliations:** ^1^ School of the Environment University of Windsor Windsor Ontario Canada; ^2^ Environment and Climate Change Canada Canada Centre for Inland Waters Burlington Ontario Canada

**Keywords:** bacteria, biofilm, community assembly, freshwater bacteria, microbial communities

## Abstract

Despite constituting an important component of freshwater ecosystems, biofilm assemblages have remained relatively understudied compared to plankton, especially in freshwater systems such as the western basin of Lake Erie (WBLE). This study therefore aimed to elucidate temporal and vertical shifts of microbial communities of planktonic and biofilm growth on artificial substrates in the WBLE water column at discrete depths, investigating the overlap of shared taxa between community types. Sequencing of the 16S rRNA gene revealed concurrent biofilm‐plankton samples shared a low percentage (~10%) of amplicon sequence variants (ASVs) indicating distinct communities between free‐living and substrate‐attached bacteria. Plankton communities did not significantly differ between surface and bottom depths (1 and 8 m), whereas biofilm communities differed between upper (1–4 m) and lower (5–8 m) water columns. Temporal variation in community composition was observed in biofilm, with early periods (June–July) showing significant dissimilarity followed by compositional convergence in late summer onwards (August–October). With the expansion of artificial infrastructure in aquatic systems, there is novel substrate material to observe spatiotemporal patterns of microbial colonisation throughout the pelagic zone. These results demonstrate the complexity of bacterial biofilm communities from plankton in freshwater, providing insight into microbial assembly through temporal succession and across depth.

## Graphical abstract

Biofilm and planktonic bacterial communities in Lake Erie's western basin show distinct compositions, with only ~10% shared ASVs. While plankton communities remain consistent across depths, biofilms exhibit depth‐ and time‐dependent variability, with early‐summer communities differing before converging in late summer. These findings highlight the complexity of microbial colonization on artificial substrates from surrounding plankton in freshwater ecosystems.

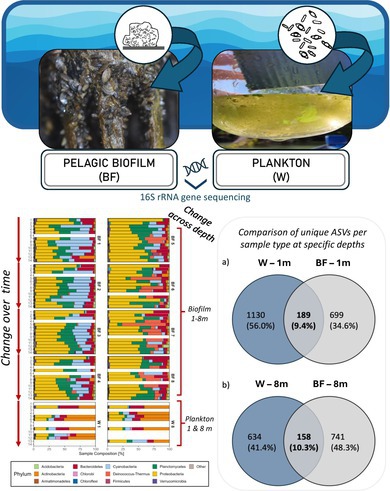

## 1 Introduction

Anthropogenically driven factors such as sediment and nutrient loading, climate change, and invasive species strongly influence the composition of microorganisms in freshwater systems (Berry et al. 2017; O'Donnell et al. 2023). As a result, within the smallest of the Laurentian Great Lakes (*hereafter* Great Lakes), the western basin of Lake Erie (WBLE) has undergone extensive ecosystem shifts in its microbial communities (i.e., bacteria, eukaryotes, algae; Allinger and Reavie 2013). Nutrient runoff, particularly phosphorus and nitrogen from agricultural and wastewater sources, fuelled the proliferation of phytoplankton, facilitating eutrophic conditions and subsequently harmful algal blooms (HABs) in western Lake Erie (Jankowiak et al. 2019). The Great Lakes Water Quality Agreement (1972–current) set a precedent by explicitly targeting phosphorus loading to address nutrient concentrations in the Great Lakes. Despite initial success, characterised by reductions in phosphorus levels (De Pinto, Young, and McIlroy 1986), the frequency and duration of algal blooms have not shown a corresponding decline in the last few decades (1990–present; Watson et al. 2016). The introduction of invasive zebra ( *Dreissena polymorpha* ) and quagga ( *Dreissena rostriformis bugensis* ) mussels (*hereafter* dreissenid mussels, collectively) in the late 1980s dramatically altered nutrient cycling and sequestration (Makarewicz, Bertram, and Lewis 2000); water clarity (Barbiero and Tuchman 2004); and phytoplankton community structure (Makarewicz, Lewis, and Bertram 1999) in the Great Lakes.

Microbial communities of freshwater systems have been identified as biological indicators of water quality and ecosystem resilience (Hellawell 2012; Sagova‐Mareckova et al. 2021), where microorganisms exist in two key life strategies: free‐living single‐cell plankton and community‐aggregated assemblages (*hereafter* biofilm). The process of biofilm formation is initiated through planktonic microbial adhesion, often to a surface or substratum, forming a heterogeneous multi‐species matrix within an extracellular polymeric substance (Wimpenny, Manz, and Szewzyk 2000; Dang and Lovell 2016). Mechanisms driving biofilm development have been identified through a range of stochastic (Brislawn et al. 2019); abiotic (e.g., light; Rao et al. 1997; Sekar et al. 2002); and biotic processes, unique across aquatic systems.

In the WBLE, planktonic communities and consequential changes have been well documented in previous decades, finding distinct spatiotemporal variation among communities (O'Donnell et al. 2023). Seasonally, planktonic microbial communities are dominated by strains of cyanobacteria during summer months (Matteson et al. 2011; Wilhelm et al. 2014). Cyanobacteria in the WBLE have been of particular concern due to their contribution to cyanobacteria‐dominated HABs during warm, eutrophic conditions (Jankowiak et al. 2019). Studies assessing plankton community variation across depths appear absent in the WBLE, likely due to its shallow bathymetry and well‐mixed nature. However, depth distinction of plankton communities has been observed across the Great Lakes in deeper, stratified (> 40 m) areas (Paver, Newton, and Coleman 2020). In comparison, localised microbial biogeography has been observed among WBLE sediment profiles at depths less than 1 m apart (Vadeboncoeur et al. 2014; Tedeschi and Chow‐Fraser 2021), resulting in substantial variation between proximate communities. Studies on microbial assemblages in the WBLE are often limited to the benthos and pertain to organic substrates (i.e., rock, sediment) as the attachment surface. Yet, as an important constituent of lake ecosystems, pelagic biofilms and biofilm‐focused studies in the Great Lakes are needed to assess seasonal and depth‐related variation in a changing environment.

It may be expected that a microbial assemblage (i.e., biofilm) is strongly influenced by the planktonic free‐living microbial community in the surrounding system. For example, in Lake Baikal, biofilms shared analogous taxonomic components to those of plankton, though composition differed (Parfenova, Gladkikh, and Belykh 2013). Studying biofilm community composition can therefore allow one to infer potential microbial interactions which cannot be deduced from free‐living communities. Yet, comparative analyses between plankton and biofilm communities in the Lake Erie water column appear to be absent, providing a considerable knowledge gap regarding the taxonomic composition of biofilms and subsequent connection to planktonic communities, and therein assessing colonisation assembly and growth patterns of bacterial taxa. Further, due to their immobilised nature, microbes within biofilms are arguably more susceptible to vertical environmental gradients within the water column (i.e., dissolved oxygen, light intensity, temperature, etc.) than planktonic microbes. Therefore, the composition of biofilms at varying depths throughout the water column in a well‐mixed system may reveal microbial responses to changes in water quality conditions at fixed depths.

Here, we used high‐throughput sequencing of the 16S rRNA gene to identify complex bacterial community dynamics of freshwater biofilms from late spring to early fall in the WBLE. This paper investigates biofilm community colonisation, assembly, and succession across the water column and over 139 days of exposure growth in the WBLE, with emphasis on bacterial colonisation of an artificial habitat. We further compared the overlap between taxa identified from two microbial community types (plankton and biofilm), where we hypothesised: (i) the microbial communities inhabiting the biofilm (substrate‐attached) will exhibit a high percentage of shared amplicon sequence variants (ASVs) with the microbial community present in planktonic samples; (ii) biofilm microbial communities will undergo temporal variations over the study duration, with increased diversification from the planktonic community with time; and (iii) biofilm microbial communities will exhibit changes in composition and structure along vertical gradients within the water column. These data provide a new and better understanding of the dynamics driving community assembly and succession of substrate‐attached bacteria, and ultimately ecosystem function and health of the WBLE and the Great Lakes in general.

## 2 Methodology

### 2.1 Study Sites and Sample Collection

This study focused on one site location (42°01′.332 N, 82°40′.188 W) towards the northern shore of the WBLE, deployed from 22 May 2022 to 22 October 2022, a total of 153 days. On‐site, an anchored line of black acetal units (Delrin acetal homopolymer; Innovasea, Halifax, NS, Canada) was suspended in the water column at 1 m increments from lake surface to bottom, providing a substrate for biofilm colonisation. The acetal material of the units is identical to the material used for real‐time environmental monitoring instrumentation within the same area (raeon.org), replicating biofouling effects on the artificial substrates. In the context of this study, biofouling is defined as the unintended accumulation of microorganisms, plants, and/or invertebrates onto a submerged substrate (Flemming 2002), from which we collected biofilm material. The utilisation of semi‐permanent aquatic infrastructures creates new habitat in the pelagic zone for microbial taxa to colonise, where there were previously no natural substrates to do so. Following deployment, substrate units were left submerged continuously, only removing them during biofilm sample collections (< 15 min on each collection date) from fouling growth accumulated on the artificial surface. Substrate units were sampled every other week (May–June, October) and weekly (July–September), with the first sample collection 24 days after initial line deployment (15 June 2022) (Table 1). In August, 3 days of consecutive sampling were performed to observe fine‐scale (daily) differences in community composition of established biofilms. The number of days since the substrate's initial deployment to allow for biofilm colonisation is hereafter referred to as exposure time. The biofilm collections were taken by placing a 2 cm^2^ metal template on the undistributed surface of each unit and mechanically scraping duplicate squares completely of fouling growth using metal spatulas, nylon brushes, and MilliQ H_2_O rinsed into 100 mL glass amber bottles. To avoid harvesting biofilm from the same area during the next sampling collection, the scraped area was marked with liquid correction fluid. Between each sampling occurrence, biological cross‐contamination was prevented by cleaning equipment (e.g., utensils, collection bottles) with 95% ethanol and MilliQ rinses prior to and between use. Adjacent water samples (volume = 250 mL) were taken at two depths, 1 m below surface (hereafter surface) and 1 m above lake bottom (hereafter bottom) using a metered horizontal Van Dorn water sampler. Water collections were collected in an acid‐washed HDPE amber bottle, rinsed three times prior with lake water. All samples were kept dark and on ice until sampling processing within 12 h of collection. A total of 112 biofilm and 20 plankton samples were collected for eDNA sequencing and biomass weighing, respectively.

**TABLE 1:** Sampling dates for biofilm and water collection sampling in the western basin of Lake Erie in 2022. Exposure time indicates the duration of time (in days) that the substratum has been deployed for microbial adhesion and development in the lake.

Sampling event	Sampling date	Exposure time (in days)
1	June 15	24
2	July 2	41
3	July 15	54
4	July 26	65
5	August 5	75
6	August 9	79
7	August 10	80
8	August 11	81
9	August 17	87
10	August 23	93
11	September 4	105
12	September 8	109
13	September 23	124
14	October 8	139

### 2.2 Biomass Weighing

Biomass weighing was performed gravimetrically, by determining the wet and dry mass of samples. Biofilm and aliquots of bulk water samples were filtered through a vacuum pump filtration apparatus onto pre‐weighted 0.7 μm glass fibre filter papers (GF/F; 47 mm diameter; Whatman). MilliQ water was used to rinse the sample through the filter paper as needed, and the wet weight of the sample and filter paper was recorded. Samples were then placed into a 50°C drying oven for a minimum of 24 h to remove water content. Following the drying period, samples were removed from the oven and acclimated at room temperature for 1–2 h before being re‐weighed. Total biomass weight was expressed by subtracting the initial weight of the dry filter paper from total dry weight. Total biomass of the sample was considered as the masses of all biological materials unable to pass through the GF/F paper, as a measure of combined autotrophic–heterotrophic mass. Following weighing, a total of 70 samples having insufficient material (≤ 0.0 g) were omitted from further analysis.

### 2.3 eDNA Metabarcoding

To prepare samples for downstream molecular analysis, biofilm and plankton samples were filtered with a vacuum pump onto polyethersulphone filters (PES/F; 0.2 μm porosity; 47 mm diameter; Millipore), rinsing down with MilliQ as needed. Equipment was rinsed with 95% EtOH and MilliQ between each sample filtration and PES filters were stored at −20°C until DNA extraction.

For biofilm samples, DNA was extracted using the DNeasy PowerBiofilm Isolation Kit (QIAGEN, Germantown, Maryland; *hereafter* PowerBiofilm), while plankton DNA was extracted from water samples using the DNeasy PowerWater Kit (QIAGEN), following the manufacturer's protocol. For filtered biofilm samples containing relatively high biomass, a portion of biofilm was removed from the membrane filter using sterilised tweezers and weighed (0.05–0.2 g). For low biomass samples (< 0.2 g), filters were sectioned into pieces using scalpels and added directly to bead solution tubes from the PowerBiofilm Kit.

Extracted DNA samples were sent to the Integrated Microbiome Resource at Dalhousie, Halifax, Nova Scotia, for library preparation and sequencing of the universal 16S rRNA gene sequence (V4‐V5 regions), using the 515FB (5′‐GTGYCAGCMGCCGCGGTAA‐3′) and 926R (5′‐CCGYCAATTYMTTTRAGTTT‐3′) primer pair on an Illumina MiSeq platform (~300 bp).

For each sample collection event, one control PES filter paper was processed following the filtration and extracted methods as outlined above to test for potential contamination. None of the controls showed PCR amplification, showing that negligible to non‐existent external contamination was introduced during sample processing.

### 2.4 Bioinformatic Analysis

All statistical analyses were conducted using R Studio (v.4.2.2; R Core Team 2022), via high‐performance computational environments of Shared Services Canada (Dorval, Quebec; Crevecoeur et al. 2023). Primer removal was performed using Cutadapt (v.4.7; Martin 2011). Sequence truncation, trimming, chimera removal, and dereplication were performed following the Divisive Amplicon Denoising Algorithm (DADA2, v.1.8.0) analysis pipeline (Callahan et al. 2016), chosen for its greater sensitivity and resolution than other bioinformatic pipelines (Prodan et al. 2020). DADA2 methods followed details previously outlined in Crevecoeur et al. (2023). Visual assessment of sequence quality plots determined truncation lengths at 220 bp for forward reads and 175 bp for reverse reads. Taxonomy of ASVs was assigned using the SILVA reference database (Quast et al. 2012) and the BOLD database (Ivanova et al. 2019). Herein, we employ the primary database SILVA for taxonomic identification across all taxa, with the exception of ASVs within the phylum Cyanobacteria. The BOLD database was used to identify Cyanobacteria ASVs, as BOLD is a specified reference database constructed using sequences derived from cyanobacterial and algal cultures in the Great Lakes (Ivanova et al. 2019).

Resulting ASV tables were further processed as phyloseq objects (phyloseq package; v.1.42.0; McMurdie and Holmes 2013). Taxa identified as Archaea (kingdom), Eukaryota (kingdom), or Chloroplast (class) were subsequently removed from the data. At the phylum level, the community composition of samples was visualised as stacked barplots drawn with ggplot2 (v.3.5.0, Wickham 2016), where unique ASVs pertaining to less than 1% of the total sample composition were re‐classified as “Other.” This was similarly applied to taxa within the phylum Cyanobacteria, identified at the genus level by the BOLD database. Venn diagrams illustrating the shared and unique ASVs within biofilm and plankton were constructed with ggvenn (v.0.1.10, Yan 2023), filtered to include only samples collected concurrently at both surface and bottom depth.

Prior to statistical analyses, samples were rarefied to the lowest sample size of 1869 reads by 100 iterations of rarefaction over all samples. The community data were rarefied using *rarefy*_*even*_*depths* (phyloseq), with the random seed value set to 1 for reproducibility. Alpha (observed richness, Shannon's diversity index, Chao1 richness) was calculated using the *estimate_richness* function (phyloseq) on rarefied data. Differences in Shannon's diversity index was statistically assessed between sample types (biofilm vs. plankton) and across sampling events (within each sample type‐depth) using independent non‐parametric Kruskal–Wallis tests (*krusksal*.*test* function, stats package, v.4.2.2, R Core Team). Between plankton and biofilm samples, Spearman's non‐parametric correlation was applied to compare sample biomass against Shannon's diversity index and Chao1 richness, respectively.

Permutational multivariate analyses of variance (PERMANOVA) were conducted to compare communities between sample types (biofilm vs. water) at concurrent depths (surface and bottom), using the *adonis2* function (vegan package, v.2.6‐4, Oksanen 2022) with 999 permutations. PERMANOVAs were also run to observe (i) differences in plankton communities between the two sampling depths, and (ii) differences in biofilm communities between depths (1–8 m) across sampling events. This analysis included interaction between depth and sampling event as predictors, but removed interaction if deemed non‐significant. Pairwise comparison was determined using the *pairwise*.*Adonis2* function (pairwiseAdonis package, v. 0.4.1, Martinez Arbizu 2017) to assess which biofilm communities were significantly different between depth and time (sampling event). Non‐metric multidimensional scaling (NMDS) plots were created through Bray–Curtis dissimilarity matrix calculations with the *ordinate* and *plot_ordination* functions (phyloseq) differences in beta diversity of ASV abundance between samples.

## 3 Results

Following sequencing, four biofilm samples (4/107, 3.74%) and seven plankton samples (7/19, 36.8%) exhibited weak or failed amplification and were omitted from subsequent analysis. The successful 103 biofilm and 12 plankton samples yielded a total of 695,980 sequence reads of 6052 ASVs. In all, 5330 and 1528 unique ASVs were returned in biofilm and plankton samples, respectively. Taxonomic assignment based on the training database (refer to methods; SILVA, BOLD) identified sequences refined to the Bacteria domain, encompassing 34 phyla, 230 families, and 479 genera.

**FIGURE 1:**
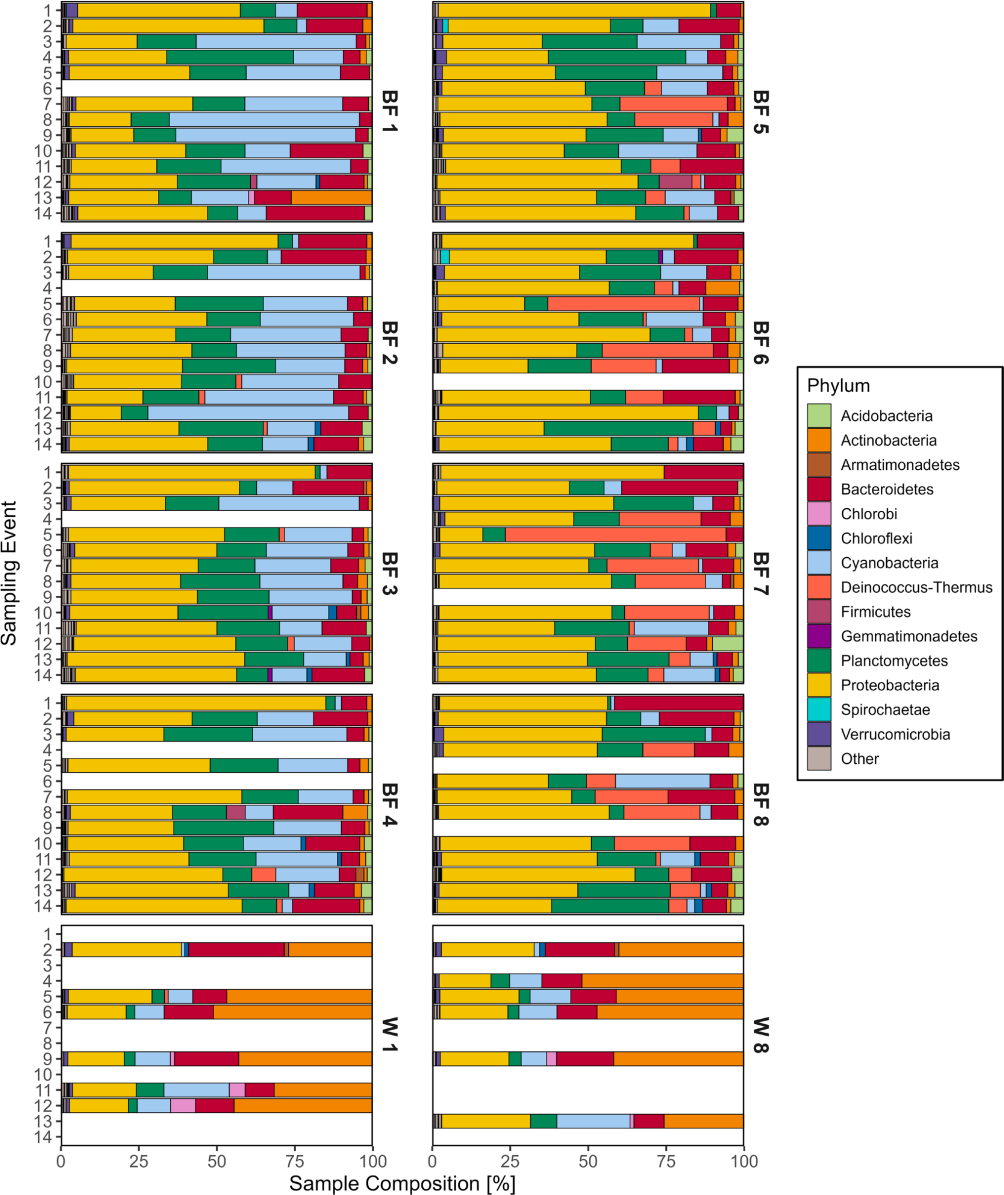
Community composition of biofilm (BF) and water (W) samples identified at phylum level at all depths (1–8 m) over the sampling period in WBLE. Taxa consisting of an abundance < 1% of total sample composition and unknown ASVs are grouped into “Other.”

The phyla Proteobacteria dominated biofilm samples, while plankton were primarily comprised of Actinobacteria (Figure 1). Proteobacteria (43.9%), Planctomycetes (16.4%), and Cyanobacteria (13.4%) were on average the most abundant phyla of all biofilm samples (*n* = 103). In contrast, the three most abundant phyla on average for water samples (*n* = 12) were Actinobacteria (40.3%), Proteobacteria (23.8%), and Bacteroidetes (12.8%). Of the 34 phyla identified, 5 phyla were observed within all samples (*n* = 115) over the study duration: Actinobacteria, Bacteroidetes, Cyanobacteria, Planctomycetes, and Proteobacteria—consistent with the most abundant phyla for biofilm and plankton samples as previously described. The presence and absence of ASVs varied temporally, such as phyla Deinococcus‐Thermus first appearing in the biofilms after 65 days of exposure, with depths ≥ 5 m all having > 1% of total abundance (Figure 1). Additionally, the relative abundance of taxa varied with depth, as there was an evident decrease in the presence of Cyanobacteria in biofilm microbial communities at sampling depths greater than 4 m. An opposite trend was observed for Cyanobacteria in the water, with the greatest abundance of Cyanobacteria comprising 23.4% of the free‐living community at 8 m on sampling event 13 (Figure 1).

**FIGURE 2:**
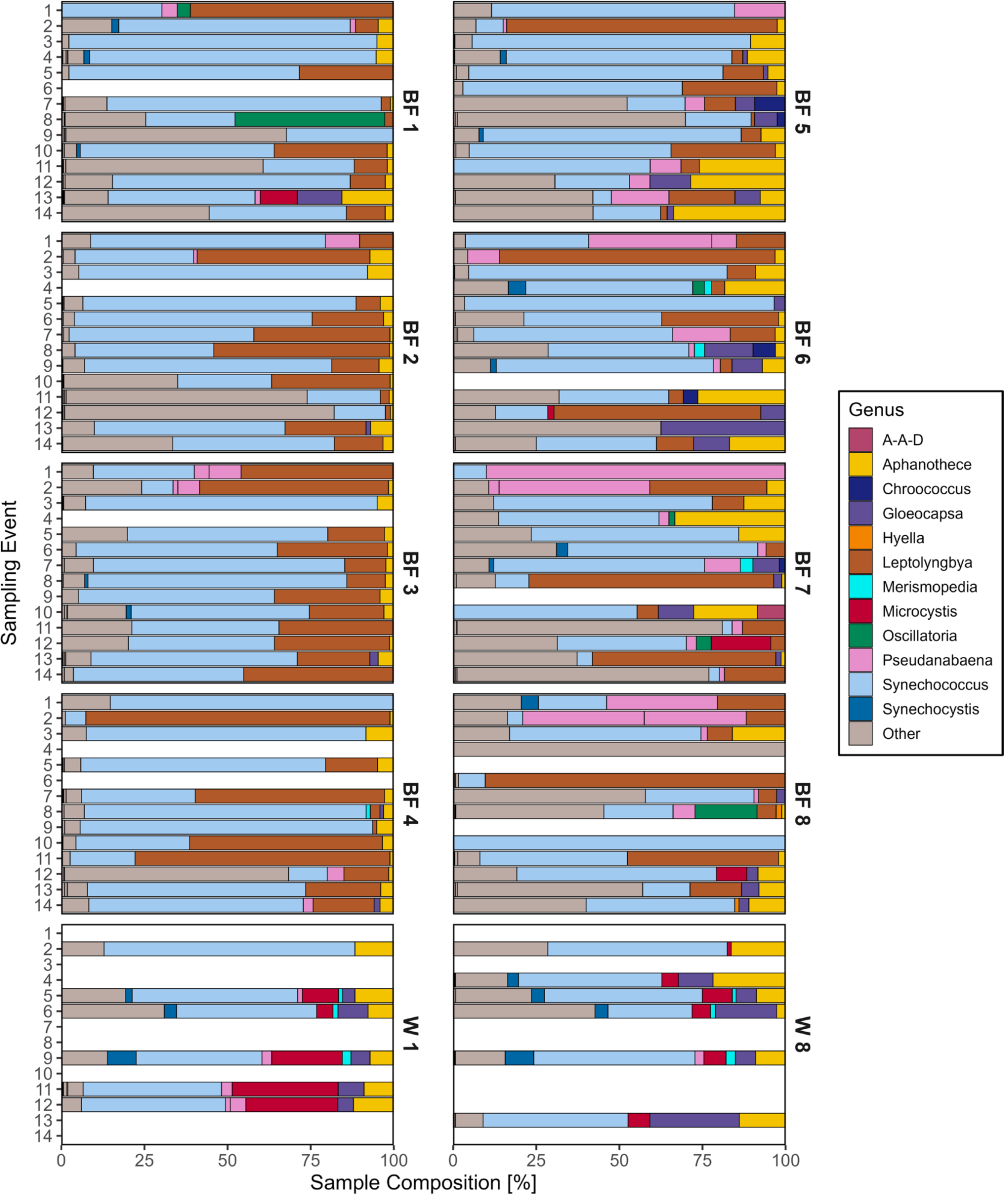
Community composition of biofilm (BF) and water (W) samples at all depths (1–8 m) over the sampling period. Identified at the genus within the Cyanobacteria phylum. Taxa consisting of an abundance < 1% of the total sample composition and unknown ASVs are grouped into “Other.”

For both sample types, *Synechococcus* was the most prominent genus within the Cyanobacteria phylum, observed in all planktonic communities (*n* = 12/12) and nearly all (*n* = 99/103) biofilm communities (Figure 2). *Synechococcus* comprised 7.8% ± 9.5% of overall composition and 48.8% ± 26.1% of total Cyanobacteria in substratum‐attached communities, whereas free‐living *Synechococcus* contributed 4.59% ± 2.8% overall composition and 46.0% ± 11.7% of total Cyanobacteria. *Leptolyngbya* was similarly found in a majority of biofilm samples (*n* = 88/103), but was only detected in one planktonic sample on the second‐last sampling date (23 September) at a low abundance of < 1% for both total microbial community and Cyanobacteria proportion. In contrast, *Leptolyngbya* contributed 3.6% ± 4.9% of overall microbial community composition and 23.2% ± 23.5% of Cyanobacteria in biofilm. *Microcystis* was found in 13.9% (16/115) of all samples, primarily plankton samples (11/16)—with the highest relative abundance of *Microcystis* found in surface plankton samples, increasing over the study duration (Figure 2). *Microcystis* was only found at extremely low abundance in the biofilm samples, comprising < 0.12% of overall microbial composition and < 11.2% of Cyanobacteria proportion. Furthermore, *Microcystis* in biofilm communities was only detected at two sampling events (8 and 23 September), 109 days after substratum deployment. In comparison, *Microcystis* was found in the planktonic community in the first successfully sequenced water sample at 8 m, albeit at an extremely low abundance of 0.02% of the overall microbial community.

**FIGURE 3:**
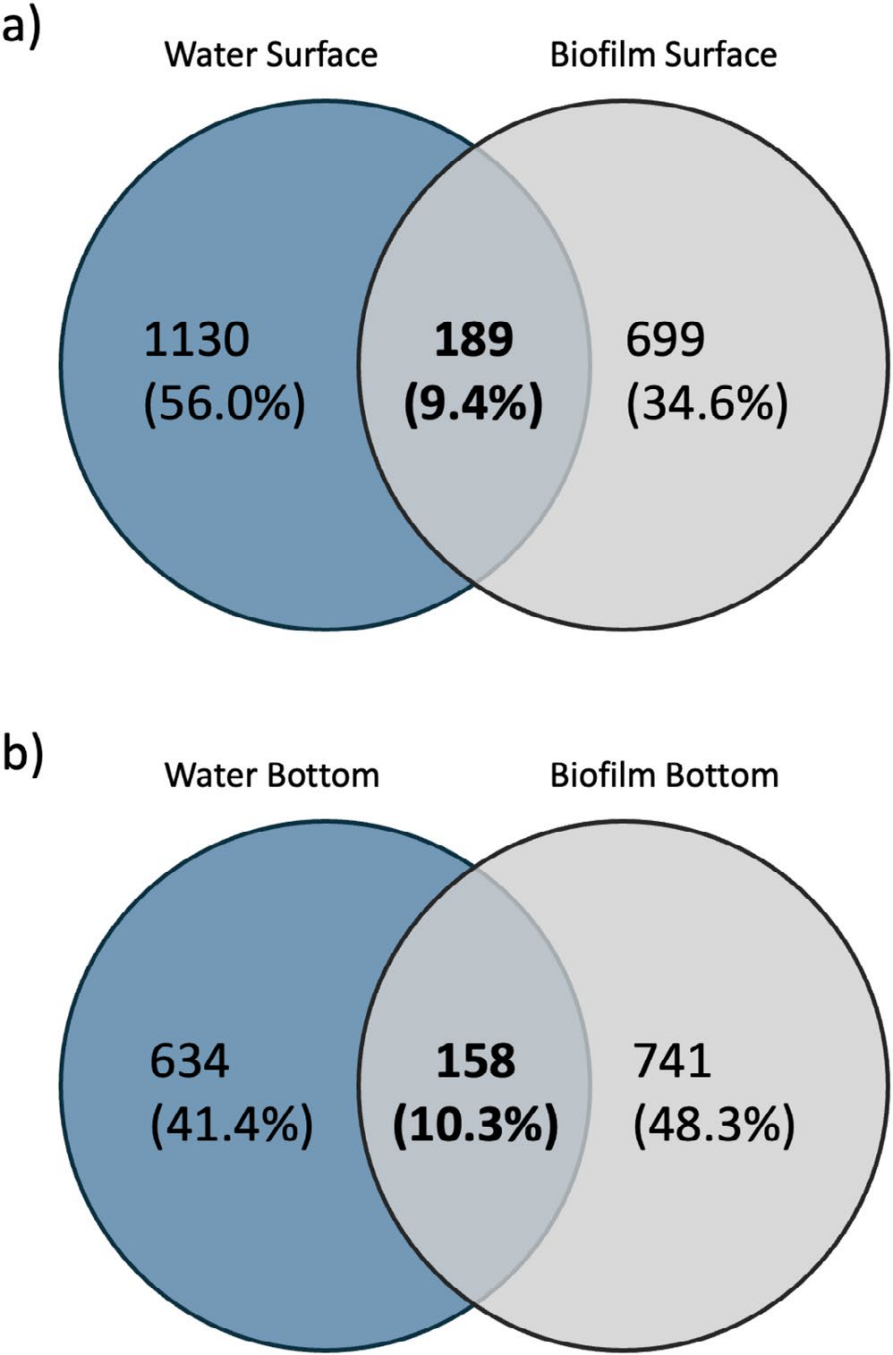
Venn diagram displaying the number and total proportion of all unique ASVs between water and biofilm samples at (a) surface—1 m and (b) bottom—8 m. Numbers within each section indicate the count of unique and overlapping ASVs between sample types, with the total proportion of all detected ASVs displayed. Only sampling events with successful sequencing from both water and biofilm samples were included in the analysis.

The comparison of biofilm and plankton samples collected concurrently revealed the intersection of ASVs between the two sample types. Among the total of 6052 unique ASVs sequenced, 806 (13.3%) were present in both plankton and biofilm. While 4524 (74.8%) were exclusive to the biofilm communities, 722 ASVs (11.9%) were exclusive to planktonic communities. Specifically, the degree of ASV overlap was quantified as 9.4% for surface and 10.3% for bottom sample type comparison for concurrently sampled biofilm–plankton communities (Figure 3). Of the 6052 ASVs sequenced, 4385 ASVs were retained following rarefaction used to calculate richness indices and statistical testing (Supporting Information 1). Taxonomic richness and diversity were similar between the two community types (Kruskal–Wallis; *p* > 0.1), with no clear trends over time. Both Shannon's diversity index and Choa1 (Supporting Information 2) were significantly correlated with biofilm total biomass, with a weak–moderate positive correlation (Supporting Information 3 and 4). However, no significant correlation was identified between diversity indices and biomass for plankton samples (Supporting Information 3 and 4).

**FIGURE 4:**
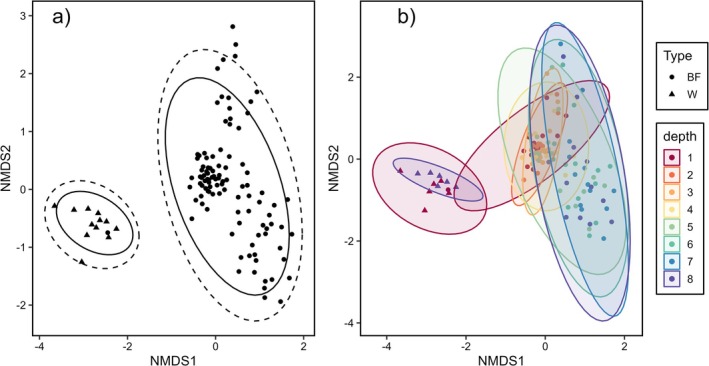
Nonmetric multidimensional scaling (NDMS) ordination plot of biofilm (BF) and water (W) communities from sampling of June to October 2022. Stress = 0.134, with Bray–Curtis abundance‐weighted dissimilarity metrics. On the left (a), dashed lines represent normal distribution, while the solid lines represent t‐distribution between sample types. On the right (b), solid lines represent normally distributed data between sample types by depth.

The microbial community composition significantly differed among the two sample types (plankton vs. biofilm, Table 2; PERMANOVA, *p* < 0.01), with dissimilarity between types demonstrated by distinct spatial separation in ordination space (Figure 4, stress = 0.1134). There was observed clustering of sample types, with the planktonic microbial communities having no statistically significant difference between surface and bottom (Table 2; PERMANOVA, *p* = 0.622). However, biofilm communities sampled along the depth gradient were dissimilar throughout the study duration and demonstrated relatively inconsistent temporal patterns. Further PERMANOVA revealed significant effects of temporal and vertical distribution on the composition of microbial biofilm communities (Table 4). Differences between sampling events explained a substantial proportion of the observed variability (*R* ^2^ = 0.35, *pseudo*‐F = 4.8014, *p* < 0.001). Similarly, variation in water column depths significantly influenced biofilm composition, albeit to a lesser extent (*R* ^2^ = 0.18, *pseudo*‐F = 4.5166, *p* < 0.001). Pairwise comparison of biofilm communities across sampling events indicated a distinct temporal relationship irrespective of depth, with the greatest degree of community dissimilarity expressed from early (< 30 days) sampling into the study (Table 3). Biofilm communities from the first three sampling events were significantly different from all other sampling events (June 15–July 15; 54 days of exposure; Table 3). From late July to late August (sampling events 4–9, 65–87 days of exposure), comparisons between sample events were variable in significance, possibly representing a transitional phase during biofilm development. Interestingly, at fine‐scale sampling (events 6–8; August 9–11), there was no significant difference between the biofilm communities sampled along the entire depth gradient. Late August to early October (sampling events 10–14, 93–139 days on exposure) then showed a reconvergence in biofilm similarity (Table 3), where the final four sampling events (September 4–October 8), showed non‐significant pairwise variation between the biofilm communities. Along the depth gradient, there was obvious clustering of biofilm samples at groupings of 1–4 m and 5–8 m, respectively (Figure 4). PERMANOVA pairwise comparison of biofilm taxonomic profiles supported NMDS clustering by depth (Table 4), revealing discrete groupings of non‐significant pairs between 1–4 and 5–8 m. Across these two depth groups, pairwise comparison between biofilm communities of 4 and 5 m depths indicated a non‐significant *p*‐value of 0.059.

**TABLE 2:** Permutational multivariate analysis of variance (PERMANOVA) results based on Bray–Curtis dissimilarity distance matrix between samples to compare community composition between groups in WBLE (Target). Significant values (*p* < 0.05) shown in bold. For a balanced comparison between plankton and biofilm communities (at either surface or bottom), samples were filtered to include only sampling events that include successfully sequenced data of both sample types.

Target		Df	SS	*R* ^2^	F	Pr(> F)
Plankton surface ~ biofilm surface	Type	1	1.66	0.48	8.32	**0.002**
	Residual	9	1.80	0.52		
	Total	10	3.50	1.00		
Plankton bottom ~ biofilm bottom	Depth	1	1.41	0.46	6.70	**0.008**
	Residual	8	1.68	0.54		
	Total	9	3.08	1.00		
Plankton surface ~ plankton bottom	Depth	1	0.08	0.06	0.62	0.731
	Residual	10	1.33	0.94		
	Total	11	1.42	1.00		
Biofilm all ~ event + depth	Event	13	10.98	0.32	3.90	**0.001**
	Depth	7	5.52	0.16	3.64	**0.001**
	Residual	82	17.8	0.52		
	Total	102	34.3	1.00		

**TABLE 3:** Bray–Curtis PERMANOVA pairwise multilevel comparison of biofilm samples with change over 14 sampling events in WBLE. Bolded *p*‐values represent significant comparisons of *p* > 0.05. Italicised cells represent the pairwise comparisons of the fine‐scale sampling period. Column and row names represent the sampling date.

	1	2	3	4	5	6	7	8	9	10	11	12	13
1													
2	**0.002**												
3	**0.001**	**0.001**											
4	**0.002**	**0.002**	**0.030**										
5	**0.001**	**0.001**	**0.021**	0.141									
6	**0.001**	**0.001**	**0.009**	0.089	0.466								
7	**0.001**	**0.001**	**0.004**	0.234	0.323	*0.28*							
8	**0.001**	**0.001**	**0.007**	0.256	0.298	*0.198*	*0.841*						
9	**0.001**	**0.001**	**0.001**	**0.049**	0.211	0.173	0.142	0.102					
10	**0.001**	**0.001**	**0.002**	**0.048**	0.079	0.122	0.153	0.124	0.204				
11	**0.001**	**0.001**	**0.001**	**0.030**	0.56	0.065	0.081	0.116	0.205	0.440			
12	**0.001**	**0.001**	**0.001**	**0.016**	**0.033**	**0.019**	0.081	0.098	0.074	0.171	0.625		
13	**0.002**	**0.001**	**0.002**	**0.023**	**0.021**	**0.021**	**0.045**	0.063	**0.038**	0.091	0.204	0.435	
14	**0.001**	**0.001**	**0.001**	**0.011**	**0.008**	**0.012**	**0.042**	**0.030**	**0.010**	0.064	0.150	0.230	0.509

**TABLE 4:** Pairwise multilevel comparison of PERMANOVA (post hoc) of biofilm samples with change in substrate depth (1–8 m) over the study period. Bolded *p*‐values represent significant comparisons. Column and row names represent sample depth in the water column.

	1	2	3	4	5	6	7
1							
2	0.985						
3	0.152	0.571					
4	0.117	0.257	0.660				
5	**0.002**	**0.001**	**0.004**	0.083			
6	**0.001**	**0.001**	**0.001**	**0.002**	0.499		
7	**0.001**	**0.001**	**0.001**	**0.001**	0.078	0.905	
8	**0.001**	**0.001**	**0.001**	**0.001**	0.67	0.678	0.908

## 4 Discussion

This study presents a novel contribution to the understanding of freshwater biofilm community dynamics through summer and early autumn (May–October) by assessing temporal and vertical variation within the water column of Lake Erie. While long‐term or permanent anthropogenic structures are relatively uncommon in offshore areas of the Great Lakes, there has been an increasing number of long‐term instrumentation fixtures (i.e., buoys, sub‐surface moorings) deployed to monitor and record scientific data such as water quality and meteorological (RAEON, GLOS, NOAA). Introducing new artificial substrates in pelagic areas of the Great Lakes creates novel space for microbial colonisation and assembly across temporal and spatial scales, which we investigate here. This research presents the first insight into bacterial biofilm community assembly and colonisation of artificial substrate throughout the WBLE water column.

Contrary to expectations, biofilm communities did not exhibit a high percentage of shared ASVs when compared to concurrently water‐sampled plankton, demonstrating that planktonic and substrate‐attached biofilm communities differed in Lake Erie. Community type (plankton vs. biofilm) accounted for 46%–48% of bacterial variation observed. Distinct taxa between free‐living and attached communities have been previously reported in aquatic systems, with attached communities associated with particle (Mou et al. 2013; Mohit et al. 2014; Urvoy et al. 2022), organic (Brablcová et al. 2013), and artificial (Miao et al. 2021) substratum types. Similar to this study, Hajibabaei et al. (2019) evaluated the relatedness of paired benthos and water samples in open‐water wetlands, which resulted in plankton yielding “watered‐down biodiversity” relative to benthic communities. Under certain assumptions, these findings of biofilm samples containing more unique ASVs than plankton samples are evidence of weakly competitive planktonic‐state taxa (i.e., found in low abundance) persisting in detectable abundance in biofilms. In marine systems, this perspective has been supported by short‐term studies, finding that while pioneering biofilm populations (0–9 h of colonisation) were similarly comprised of taxa in the surrounding water, successional biofilm communities (9–24 h of colonisation) exhibited substantial community shifts, a selective result of dominance by rapid‐growth surface‐colonising taxa over pioneering populations, often comprised of opportunistic, random colonisers (Lee et al. 2008). However, we further posit that taxa found exclusively in biofilm are constituents of the microbenthos. Microbes near and within lake sediment have been shown to favour biofilm structure (Sentenac et al. 2022), forming complex symbiotic relationships with the non‐microbial benthos. If the taxa found in biofilm are partial to community aggregates (i.e., biofilm), resuspension or dispersal from the benthos into the water column may result in these taxa occupying biofilm structures throughout the pelagic zone but require additional study.

The prediction that biofilm communities would significantly change over exposure time (i.e., number of days after substratum deployment) was supported by findings that biofilm communities were initially dissimilar (≤ 54 days), then similar for the remainder of observations. However, there was no observable temporal variation between biofilm and plankton communities concerning alpha diversity, indicating that diversity was relatively stable (Shannon index; Supporting Information 5). The first three sampling events taken 24, 41, and 54 days after substrate deployment resulted in the most dissimilar biofilm community composition as compared to all other sampling events. This dissimilarity observed from the earliest biofilm samples may be attributed to short‐term succession and development of taxa associated with initial substratum‐colonisation, often called primary colonisers. Fine‐scale daily sampling, performed 79–81 days into substrate deployment, indicated similar biofilm communities over short periods, although this could be attributed to the microbial community diversity reaching an equilibrium (Jackson, Churchill, and Roden 2001). This is further supported by the convergence of biofilm communities after 93 days of substratum deployment, resulting in a slight decrease in community richness (Supporting Information 5). Brislawn et al. (2019) found that bacterial biofilm communities were significantly different between the initial and final days of substrate deployment (i.e., 8 and 79 days, respectively), such that communities exhibited abundance turnover and loss of primary colonisers, trending towards homogeneous selection over time.

As expected, depth (1–8 m below surface) significantly influenced biofilm composition, but not the plankton communities in the WBLE. Thermal stratification can result in dissimilar plankton communities relative to thermocline positioning (Cantin et al. 2011). However, this was not detected for plankton communities in this study based on the on‐site thermistor chain (Supporting Information 6). We speculate that the similarity of the planktonic communities may be a result of the polymictic mixing regime of western Lake Erie, driving compositional homogeneity. However, bacterioplankton community distinction has been observed in shallow, non‐stratified, polymictic lakes (max. depth < 20 m; Aguilar, Vila and Sommaruga 2022), emphasising the importance of sampling at multiple depths. For biofilms, depth attributed 16% of community variation, which was less than the 34% attributed to exposure time. Freshwater biofilms trend towards homogeneous composition in later stages of succession (i.e., exposure time, Brislawn et al. 2019), which can presumably explain the homogeneity of biofilm taxonomy sampled from 93 days onwards. In this study, the total range of substratum depth ranged from 1 to 8 m, at 1 m increments. Within this depth range, two distinct groupings: 1–4 and 5–8 m showed shared community compositions, likely a response to environmental conditions across depth.

Environmental biophysiochemical gradients (e.g., light intensity, dissolved oxygen, temperature, chlorophyll) cause niche resource partitioning of vertical layers that can be occupied by favouring autotrophic and heterotrophic bacteria (Bramburger and Reavie 2016). In our study, variation in temperature along the depth gradient was relatively homogeneous except for episodic (3–5 days) stratification around 7 m depth in late July (Supporting Information 6), and likely not a driver of variation in community composition by depth. Temperature did exhibit an expected seasonal trend, increasing gradually from spring to a peak in mid‐summer, followed by a gradual decrease as fall approached. Other physiochemical parameters (i.e., soluble reactive phosphorus, dissolved oxygen, turbidity) were recorded on‐site throughout the study duration, although they were not analysed further. Coupling environmental data may have provided greater insight into external factors driving community competition between community types, which should be considered for future studies. Spietz et al. ( 2015) found that bacterial community composition was responsive to seasonal hypoxia in estuaries. Benthic hypoxia is a recurrent issue in the WBLE (Watson et al. 2016), which may have profound implications for the microbial composition of communities at greater depths.

It is well‐established that microorganisms aggregate into assemblages (i.e., biofilms) for a myriad of abiotic and biotic advantages (e.g., predation protection, nutrient retention, metabolic mechanisms; Davey and O'toole 2000). However, slowed growth rates and intra‐species competition for nutrients have been emphasised as consequential trade‐offs associated with microbial aggregation (Roman and Sabater 1999). It may be important to consider that not all microorganisms are partial to participating in community dynamics. For example, photosynthetic eukaryotes (microalgae) and prokaryotes (cyanobacteria) have been shown to exhibit anti‐biofilm activity through lipid production in a clinical laboratory study (Cepas et al. 2021). Additionally, several taxa in this study (e.g., Actinobacteria, Chlorobi) were found either exclusively or in higher abundance in the water column than in biofilm, possibly exhibiting an anti‐biofilm preference.

Proteobacteria, Bacteroidetes, Actinobacteria, and Cyanobacteria have been identified as abundant proponents of free‐living bacterial communities in Lake Erie (Shahraki, Chaganti, and Heath 2021; Crevecoeur et al. 2023). Findings here were broadly consistent with previous studies with overall with phyla Actinobacteria and Proteobacteria being most abundant in plankton communities (Parfenova, Gladkikh, and Belykh 2013), where Actinobacteria comprised a minimum of 25% composition per water sample. Biofilm formation of Actinobacteria is understudied and poorly understood, especially in freshwater environments. Previous reports have established that Actinobacteria appear to favour anti‐biofilm activity, specifically through abstention from microbial adhesion and quorum sensing (Azman et al. 2019; El Othmany et al. 2021). Results coincide with such principles, as the abundance of Actinobacteria in biofilm was substantially lower than in plankton. In contrast, Acidobacteria was found exclusively in biofilm samples. Acidobacteria is a prevalent constituent of freshwater sediments across the Great Lakes (Winters et al. 2014), with findings that Acidobacteria shares a strong negative correlation with habitat pH, with an optimum around 5.5 (Jones et al. 2009). Lake Erie has recorded an average summer pH range of 8.0–8.2, even elevated to ≥ 9.2 following a *Microcystis* cHAB event (Zepernick et al. 2021). As biofilms are resistant to extreme environments, such as immoderate pH conditions (Yin et al. 2019), aggregated Acidobacteria is likely able to thrive within biofilm over the planktonic state. From these standpoints, it is possible to assume that Acidobacteria prefers protective community establishments (i.e., biofilms, sediment) than planktonic existence.

Cyanobacteria were more prevalent within the biofilm as opposed to water in WBLE, which contrasts established expectations associated with Cyanobacteria obtaining optimal growth during the planktonic cell phase (Reynolds et al. 1981). However, there was an evident increase in the proportion of Cyanobacteria observed in plankton communities at the surface over the study duration. This increase could be attributed to the increase in water temperature, as the optimal temperature range designated for cyanobacterial growth has been identified between 20°C and 30°C (Konopka and Brock 1978; Yang et al. 2020). Over the 6‐month period (May–October), the first and last months recorded the lowest monthly mean water column temperatures of 16.5°C and 17.3°C, respectively. While the mean water column temperature for the remainder of the study (June–September) was greater than the floor optimal growth range threshold of 20°C. Cyanobacteria abundance at respective depths within the biofilm remained relatively consistent; however, there was a slight increase in the 1–4 m of depth around the warmest months. Cyanobacteria relative abundance then declined towards the end of the study, associated with cooler water temperatures. Mixing regimes and water column stratification are important indicators for cyanobacterial abundance in freshwater systems (Wagner and Adrian 2009; Stockenreiter et al. 2021). Yet, our findings suggest the non‐stratified water column had negligible effects on the composition of biofilm‐aggregating Cyanobacteria. Alternatively, the higher abundance of Cyanobacteria at 1–5 m depth in biofilms may be driven by light intensity and penetration into the water column, found to result in compensatory movements to preferred lake layers by buoyant or motile Cyanobacteria (Reynolds 1987).

Within Cyanobacteria, the composition at the genera level varied between community types, although it was less dissimilar than overall communities. *Synechococcus* remained an unchanged prominent contributor to the cyanobacterial community in both biofilm and plankton across depths and study duration. The ubiquity and prevalence of planktonic *Synechococcus* sp. across the Great Lakes have been thoroughly established in recent years using molecular techniques (Ouellette, Handy, and Wilhelm 2006; Jankowiak et al. 2019; Crevecoeur et al. 2023), which revealed an underestimation bias of the picobacterioplankton by microscopic identification techniques. Over time, the relative abundance of planktonic *Synechococcus* decreased with increasing *Microcystis* relative abundance at the surface, although the genus *Synechococcus* remained more dominant than *Microcystis* across all samples. Consistently, Ye et al. (2011) observed decreasing *Synechococcus* in the *Synechococcus*:*Microcystis* ratio associated with *Microcystis*‐dominated cHABs in Lake Taihu, China, indicative of variable growth rates and temperature optima. *Microcystis* is known to form single‐species buoyant colonies in the water column, which could attribute to its low to non‐existent abundance in biofilms due to its competitive nature in the water column. While it is true in our findings that biofilm Cyanobacteria were dominated by coccoid *Synechococcus*, other coccoid Cyanobacteria taxa (e.g., *Microcystis*, *Chroococcus*, *Gloeocapsa*) were found irregularly in low relative abundance. Although taxa of the genus *Planktothrix* have been previously abundant in planktonic and bloom communities in the WBLE (Davis et al. 2015; Jankowiak et al. 2019; Crevecoeur et al. 2023), it was found only as a minor constituent (< 1% total composition, if present) in this study.

## 5 Conclusions

Distinction between planktonic and biofilm communities was evident in the shallow, warm, and polymictic WBLE system. Both community types contain unique taxa, suggesting microbial specialisation or selection processes. We established that biofilm showed compositional change with depth, at a resolution of 1 m increments. Consistent with previous studies, biofilm maturation converged in taxonomic similarity over exposure time, regardless of depth. There were distinct groupings between shallow (1–4 m) and deep (5–8 m) biofilm communities, while the continuous mixing regime of the water column resulted in similar plankton between discrete surface and bottom communities. This study allows us to unravel the complex association between bacteria in this freshwater ecosystem and better understand the factors influencing bacterial community assembly and succession in a biofilm compared to a planktonic habitat. This research provides insight into temporal patterns of selection in biofilm communities resulting in similar communities over time, with greater implications for bacterial interactions that cannot be determined from open‐water samples and improved understanding of factors influencing free‐living and substratum‐attached community assembly.

## Author Contributions

**Rylie L. Robinson:** conceptualization, investigation, writing – original draft, methodology, validation, visualization, writing – review and editing, formal analysis, software, data curation. **Aaron T. Fisk:** resources, supervision, project administration, funding acquisition, conceptualization, writing – review and editing, methodology. **Sophie Crevecoeur:** conceptualization, methodology, writing – review and editing, validation, project administration, supervision, resources, data curation, software.

## Conflicts of Interest

The authors declare no conflicts of interest.

## Supporting information


**Data S1** Supporting Information.

## Data Availability

The data that support the findings of this study are openly available in the NCBI Sequence Read Archive (SRA) at https://www.ncbi.nlm.nih.gov/sra/PRJNA1182407 reference number PRJNA1182407.

## References

[emi470079-bib-0001] Allinger, L. E. , and E. D. Reavie . 2013. “The Ecological History of Lake Erie as Recorded by the Phytoplankton Community.” Journal of Great Lakes Research 39, no. 3: 365–382.

[emi470079-bib-0070] Aguilar, P. , I. Vila , and R. Sommaruga . 2022. “Bacterioplankton Zonation Does Exist in High Elevation, polymictic Lakes.” Frontiers in Microbiology 13: 764566. 35250918 10.3389/fmicb.2022.764566 PMC8891803

[emi470079-bib-0002] Azman, A.‐S. , C.‐I. Mawang , J.‐E. Khairat , and S. AbuBakar . 2019. “Actinobacteria—A Promising Natural Source of Anti‐Biofilm Agents.” International Microbiology 22: 403–409. 30847714 10.1007/s10123-019-00066-4

[emi470079-bib-0003] Barbiero, R. P. , and M. L. Tuchman . 2004. “Long‐Term Dreissenid Impacts on Water Clarity in Lake Erie.” Journal of Great Lakes Research 30, no. 4: 557–565.

[emi470079-bib-0004] Berry, M. A. , T. W. Davis , R. M. Cory , et al. 2017. “Cyanobacterial Harmful Algal Blooms Are a Biological Disturbance to Western Lake Erie Bacterial Communities.” Environmental Microbiology 19, no. 3: 1149–1162. 28026093 10.1111/1462-2920.13640

[emi470079-bib-0005] Brablcová, L. , I. Buriánková , P. Badurová , and M. Rulík . 2013. “The Phylogenetic Structure of Microbial Biofilms and Free‐Living Bacteria in a Small Stream.” Folia Microbiologica 58: 235–243. 23129136 10.1007/s12223-012-0201-y

[emi470079-bib-0006] Bramburger, A. J. , and E. D. Reavie . 2016. “A Comparison of Phytoplankton Communities of the Deep Chlorophyll Layers and Epilimnia of the Laurentian Great Lakes.” Journal of Great Lakes Research 42, no. 5: 1016–1025.

[emi470079-bib-0007] Brislawn, C. J. , E. B. Graham , K. Dana , et al. 2019. “Forfeiting the Priority Effect: Turnover Defines Biofilm Community Succession.” ISME Journal 13, no. 7: 1865–1877. 30886318 10.1038/s41396-019-0396-x PMC6775999

[emi470079-bib-0008] Callahan, B. J. , P. J. McMurdie , M. J. Rosen , A. W. Han , A. J. A. Johnson , and S. P. Holmes . 2016. “DADA2: High‐Resolution Sample Inference From Illumina Amplicon Data.” Nature Methods 13, no. 7: 581–583. 27214047 10.1038/nmeth.3869 PMC4927377

[emi470079-bib-0009] Cantin, A. , B. E. Beisner , J. M. Gunn , Y. T. Prairie , and J. G. Winter . 2011. “Effects of Thermocline Deepening on Lake Plankton Communities.” Canadian Journal of Fisheries and Aquatic Sciences 68, no. 2: 260–276.

[emi470079-bib-0010] Cepas, V. , I. Gutiérrez‐del‐Río , Y. López , et al. 2021. “Microalgae and Cyanobacteria Strains as Producers of Lipids With Antibacterial and Antibiofilm Activity.” Marine Drugs 19, no. 12: 675. 34940674 10.3390/md19120675 PMC8709229

[emi470079-bib-0011] Crevecoeur, S. , T. A. Edge , L. C. Watson , et al. 2023. “Spatio‐Temporal Connectivity of the Aquatic Microbiome Associated With Cyanobacterial Blooms Along a Great Lake Riverine‐Lacustrine Continuum.” Frontiers in Microbiology 14: 1073753. 36846788 10.3389/fmicb.2023.1073753 PMC9947797

[emi470079-bib-0012] Dang, H. , and C. R. Lovell . 2016. “Microbial Surface Colonization and Biofilm Development in Marine Environments.” Microbiology and Molecular Biology Reviews 80, no. 1: 91–138. 26700108 10.1128/MMBR.00037-15 PMC4711185

[emi470079-bib-0013] Davey, M. E. , and G. A. O'toole . 2000. “Microbial Biofilms: From Ecology to Molecular Genetics.” Microbiology and Molecular Biology Reviews 64, no. 4: 847–867. 11104821 10.1128/mmbr.64.4.847-867.2000 PMC99016

[emi470079-bib-0014] Davis, T. W. , G. S. Bullerjahn , T. Tuttle , R. M. McKay , and S. B. Watson . 2015. “Effects of Increasing Nitrogen and Phosphorus Concentrations on Phytoplankton Community Growth and Toxicity During Planktothrix Blooms in Sandusky Bay, Lake Erie.” Environmental Science & Technology 49, no. 12: 7197–7207. 25992592 10.1021/acs.est.5b00799

[emi470079-bib-0040] De Pinto Joseph, V. , T. C. Young , and L. M. McIlroy . 1986. “Great Lakes Water Quality Improvement.” Environmental Science & Technology 20, no. 8: 752–759. 22196697 10.1021/es00150a001

[emi470079-bib-0015] El Othmany, R. , H. Zahir , M. Ellouali , and H. Latrache . 2021. “Current Understanding on Adhesion and Biofilm Development in Actinobacteria.” International Journal of Microbiology 2021: 1–11. 10.1155/2021/6637438 PMC8166509 34122552

[emi470079-bib-0016] Flemming, H.‐C. 2002. “Biofouling in Water Systems–Cases, Causes and Countermeasures.” Applied Microbiology and Biotechnology 59, no. 6: 629–640. 12226718 10.1007/s00253-002-1066-9

[emi470079-bib-0017] Hajibabaei, M. , T. M. Porter , C. V. Robinson , D. J. Baird , S. Shokralla , and M. T. G. Wright . 2019. “Watered‐Down Biodiversity? A Comparison of Metabarcoding Results From DNA Extracted From Matched Water and Bulk Tissue Biomonitoring Samples.” PLoS One 14, no. 12: e0225409. 31830042 10.1371/journal.pone.0225409 PMC6907778

[emi470079-bib-0018] Hellawell, J. M. , ed. 2012. Biological Indicators of Freshwater Pollution and Environmental Management. Springer Science & Business Media.

[emi470079-bib-0019] Ivanova, N. V. , L. C. Watson , J. Comte , et al. 2019. “Rapid Assessment of Phytoplankton Assemblages Using Next Generation Sequencing–Barcode of Life Database: A Widely Applicable Toolkit to Monitor Biodiversity and Harmful Algal Blooms (HABs).” bioRxiv, 2019‐12. 10.7717/peerj.20747 PMC12951889 41777690

[emi470079-bib-0020] Jackson, C. R. , P. F. Churchill , and E. E. Roden . 2001. “Successional Changes in Bacterial Assemblage Structure During Epilithic Biofilm Development.” Ecology 82, no. 2: 555–566.

[emi470079-bib-0021] Jankowiak, J. , T. Hattenrath‐Lehmann , B. J. Kramer , M. Ladds , and C. J. Gobler . 2019. “Deciphering the Effects of Nitrogen, Phosphorus, and Temperature on Cyanobacterial Bloom Intensification, Diversity, and Toxicity in Western Lake Erie.” Limnology and Oceanography 64, no. 3: 1347–1370.

[emi470079-bib-0022] Jones, R. T. , M. S. Robeson , C. L. Lauber , M. Hamady , R. Knight , and N. Fierer . 2009. “A Comprehensive Survey of Soil Acidobacterial Diversity Using Pyrosequencing and Clone Library Analyses.” ISME Journal 3, no. 4: 442–453. 19129864 10.1038/ismej.2008.127 PMC2997719

[emi470079-bib-0023] Konopka, A. , and T. D. Brock . 1978. “Effect of Temperature on Blue‐Green Algae (Cyanobacteria) in Lake Mendota.” Applied and Environmental Microbiology 36, no. 4: 572–576. 16345318 10.1128/aem.36.4.572-576.1978 PMC243093

[emi470079-bib-0071] Lee, J. W. , J. H. Nam , Y. H. Kim , K. H. Lee , and D. H. Lee . 2008. “Bacterial Communities in the Initial Stage of Marine Biofilm Formation on Artificial Surfaces.” Journal of Microbiology 46: 174–182. 18545967 10.1007/s12275-008-0032-3

[emi470079-bib-0024] Makarewicz, J. C. , P. Bertram , and T. W. Lewis . 2000. “Chemistry of the Offshore Surface Waters of Lake Erie: Pre‐ and Post‐Dreissena Introduction (1983–1993).” Journal of Great Lakes Research 26, no. 1: 82–93.

[emi470079-bib-0025] Makarewicz, J. C. , T. W. Lewis , and P. Bertram . 1999. “Phytoplankton Composition and Biomass in the Offshore Waters of Lake Erie: Pre‐ and Post‐Dreissena Introduction (1983–1993).” Journal of Great Lakes Research 25, no. 1: 135–148.

[emi470079-bib-0026] Martin, M. 2011. “Cutadapt Removes Adapter Sequences From High‐Throughput Sequencing Reads.” EMBnet.journal 17, no. 1: 10–12.

[emi470079-bib-0027] Martinez Arbizu, P. 2017. “pairwiseAdonis: Pairwise Multilevel Comparison Using Adonis. R.”

[emi470079-bib-0028] Matteson, A. R. , S. N. Loar , R. A. Bourbonniere , and S. W. Wilhelm . 2011. “Molecular Enumeration of an Ecologically Important Cyanophage in a Laurentian Great Lake.” Applied and Environmental Microbiology 77, no. 19: 6772–6779. 21841023 10.1128/AEM.05879-11 PMC3187120

[emi470079-bib-0029] McMurdie, P. J. , and S. Holmes . 2013. “phyloseq: An R Package for Reproducible Interactive Analysis and Graphics of Microbiome Census Data.” PLoS One 8, no. 4: e61217. 23630581 10.1371/journal.pone.0061217 PMC3632530

[emi470079-bib-0030] Miao, L. , C. Wang , T. M. Adyel , et al. 2021. “Periphytic Biofilm Formation on Natural and Artificial Substrates: Comparison of Microbial Compositions, Interactions, and Functions.” Frontiers in Microbiology 12: 684903. 34381427 10.3389/fmicb.2021.684903 PMC8350161

[emi470079-bib-0032] Mohit, V. , P. Archambault , N. Toupoint , and C. Lovejoy . 2014. “Phylogenetic Differences in Attached and Free‐Living Bacterial Communities in a Temperate Coastal Lagoon During Summer, Revealed via High‐Throughput 16S rRNA Gene Sequencing.” Applied and Environmental Microbiology 80, no. 7: 2071–2083. 24463966 10.1128/AEM.02916-13 PMC3993158

[emi470079-bib-0033] Mou, X. , J. Jacob , X. Lu , S. Robbins , S. Sun , and J. D. Ortiz . 2013. “Diversity and Distribution of Free‐Living and Particle‐Associated Bacterioplankton in Sandusky Bay and Adjacent Waters of Lake Erie Western Basin.” Journal of Great Lakes Research 39, no. 2: 352–357.

[emi470079-bib-0035] O'Donnell, D. R. , R. Briland , R. R. Budnik , S. A. Ludsin , and J. M. Hood . 2023. “Trends in Lake Erie Phytoplankton Biomass and Community Structure During a 20‐Year Period of Rapid Environmental Change.” Journal of Great Lakes Research 49, no. 3: 672–684.

[emi470079-bib-0036] Oksanen, J. 2022. “vegan: Community Ecology Package. R Package Version 2.6‐4.” Retrieved from https://CRAN.R‐project.org/package=vegan.

[emi470079-bib-0037] Ouellette, A. J. A. , S. M. Handy , and S. W. Wilhelm . 2006. “Toxic Microcystis Is Widespread in Lake Erie: PCR Detection of Toxin Genes and Molecular Characterization of Associated Cyanobacterial Communities.” Microbial Ecology 51: 154–165. 16435169 10.1007/s00248-004-0146-z

[emi470079-bib-0038] Parfenova, V. V. , A. S. Gladkikh , and O. I. Belykh . 2013. “Comparative Analysis of Biodiversity in the Planktonic and Biofilm Bacterial Communities in Lake Baikal.” Microbiology 82: 91–101.

[emi470079-bib-0039] Paver, S. F. , R. J. Newton , and M. L. Coleman . 2020. “Microbial Communities of the Laurentian Great Lakes Reflect Connectivity and Local Biogeochemistry.” Environmental Microbiology 22, no. 1: 433–446. 31736217 10.1111/1462-2920.14862 PMC6973239

[emi470079-bib-0041] Prodan, A. , V. Tremaroli , H. Brolin , A. H. Zwinderman , M. Nieuwdorp , and E. Levin . 2020. “Comparing Bioinformatic Pipelines for Microbial 16S rRNA Amplicon Sequencing.” PLoS One 15, no. 1: e0227434. 31945086 10.1371/journal.pone.0227434 PMC6964864

[emi470079-bib-0042] Quast, C. , E. Pruesse , P. Yilmaz , et al. 2012. “The SILVA Ribosomal RNA Gene Database Project: Improved Data Processing and Web‐Based Tools.” Nucleic acids research 41, no. D1: D590–D596. 23193283 10.1093/nar/gks1219 PMC3531112

[emi470079-bib-0043] R Core Team . 2022. R: A Language and Environment for Statistical Computing. R Foundation for Statistical Computing. https://www.R‐project.org/.

[emi470079-bib-0044] Rao, T. S. , P. G. Rani , V. P. Venugopalan , and K. V. K. Nair . 1997. “Biofilm Formation in a Freshwater Environment Under Photic and Aphotic Conditions.” Biofouling 11, no. 4: 265–282.

[emi470079-bib-0045] Reynolds, C. S. 1987. “Cyanobacterial Water‐Blooms.” In Advances in Botanical Research, vol. 13, 67–143. Elsevier.

[emi470079-bib-0046] Reynolds, C. S. , G. H. M. Jaworski , H. A. Cmiech , and G. F. Leedale . 1981. “On the Annual Cycle of the Blue‐Green Alga Microcystis aeruginosa Kütz. emend. Elenkin.” Philosophical Transactions of the Royal Society of London. B, Biological Sciences 293, no. 1068: 419–477.

[emi470079-bib-0047] Roman, A. M. , and S. Sabater . 1999. “Effect of Primary Producers on the Heterotrophic Metabolism of a Stream Biofilm.” Freshwater Biology 41, no. 4: 729–736.

[emi470079-bib-0048] Sagova‐Mareckova, M. , J. Boenigk , A. Bouchez , et al. 2021. “Expanding Ecological Assessment by Integrating Microorganisms Into Routine Freshwater Biomonitoring.” Water Research 191: 116767. 33418487 10.1016/j.watres.2020.116767

[emi470079-bib-0072] Sekar, R. , K. V. K. Nair , V. N. R. Rao , and V. P. Venugopalan . 2002. “Nutrient Dynamics and Successional Changes in a Lentic Freshwater Biofilm.” Freshwater Biology 47, no. 10: 1893–1907.

[emi470079-bib-0050] Sentenac, H. , A. Loyau , J. Leflaive , and D. S. Schmeller . 2022. “The Significance of Biofilms to Human, Animal, Plant and Ecosystem Health.” Functional Ecology 36, no. 2: 294–313.

[emi470079-bib-0051] Shahraki, A. H. , S. R. Chaganti , and D. Heath . 2021. “Spatio‐Temporal Dynamics of Bacterial Communities in the Shoreline of Laurentian Great Lake Erie and Lake St. Clair's Large Freshwater Ecosystems.” BMC Microbiology 21: 1–15. 34548037 10.1186/s12866-021-02306-y PMC8454060

[emi470079-bib-0073] Spietz, R. L. , C. M. Williams , G. Rocap , and M. Claire Horner‐Devine . 2015. “A Dissolved Oxygen Threshold for Shifts in Bacterial Community Structure in a Seasonally Hypoxic Estuary.” PloS one 10, no. 8: e0135731. 26270047 10.1371/journal.pone.0135731 PMC4535773

[emi470079-bib-0052] Stockenreiter, M. , J. Isanta Navarro , F. Buchberger , and H. Stibor . 2021. “Community Shifts From Eukaryote to Cyanobacteria Dominated Phytoplankton: The Role of Mixing Depth and Light Quality.” Freshwater Biology 66, no. 11: 2145–2157.

[emi470079-bib-0053] Tedeschi, A. C. , and P. Chow‐Fraser . 2021. “Periphytic Algal Biomass as a Bioindicator of Phosphorus Concentrations in Agricultural Headwater Streams of Southern Ontario.” Journal of Great Lakes Research 47, no. 6: 1702–1709.

[emi470079-bib-0054] Urvoy, M. , M. Gourmelon , J. Serghine , E. Rabiller , S. L'Helguen , and C. Labry . 2022. “Free‐Living and Particle‐Attached Bacterial Community Composition, Assembly Processes and Determinants Across Spatiotemporal Scales in a Macrotidal Temperate Estuary.” Scientific Reports 12, no. 1: 13897. 35974094 10.1038/s41598-022-18274-w PMC9381549

[emi470079-bib-0055] Vadeboncoeur, Y. , S. P. Devlin , P. B. McIntyre , and M. J. Vander Zanden . 2014. “Is There Light After Depth? Distribution of Periphyton Chlorophyll and Productivity in Lake Littoral Zones.” Freshwater Science 33, no. 2: 524–536.

[emi470079-bib-0056] Wagner, C. , and R. Adrian . 2009. “Cyanobacteria Dominance: Quantifying the Effects of Climate Change.” Limnology and Oceanography 54, no. 6part2: 2460–2468.

[emi470079-bib-0057] Watson, S. B. , C. Miller , G. Arhonditsis , et al. 2016. “The Re‐Eutrophication of Lake Erie: Harmful Algal Blooms and Hypoxia.” Harmful Algae 56: 44–66. 28073496 10.1016/j.hal.2016.04.010

[emi470079-bib-0074] Wickham, H. 2016. ggplot2: Elegant Graphics for Data Analysis. Springer‐Verlag. https://ggplot2.tidyverse.org.

[emi470079-bib-0058] Wilhelm, S. W. , G. R. LeCleir , G. S. Bullerjahn , et al. 2014. “Seasonal Changes in Microbial Community Structure and Activity Imply Winter Production Is Linked to Summer Hypoxia in a Large Lake.” FEMS Microbiology Ecology 87, no. 2: 475–485. 24164471 10.1111/1574-6941.12238

[emi470079-bib-0059] Wimpenny, J. , W. Manz , and U. Szewzyk . 2000. “Heterogeneity in Biofilms.” FEMS Microbiology Reviews 24, no. 5: 661–671. 11077157 10.1111/j.1574-6976.2000.tb00565.x

[emi470079-bib-0060] Winters, A. D. , T. L. Marsh , T. O. Brenden , and M. Faisal . 2014. “Molecular Characterization of Bacterial Communities Associated With Sediments in the Laurentian Great Lakes.” Journal of Great Lakes Research 40, no. 3: 640–645.

[emi470079-bib-0061] Yan, L. 2023. “ggvenn: Draw Venn Diagram by ‘ggplot2’. R Package Version 0.1.10.” Retrieved from https://CRAN.R‐project.org/package=ggvenn.

[emi470079-bib-0062] Yang, Z. , M. Zhang , Y. Yu , and X. Shi . 2020. “Temperature Triggers the Annual Cycle of Microcystis, Comparable Results From the Laboratory and a Large Shallow Lake.” Chemosphere 260: 127543. 32659542 10.1016/j.chemosphere.2020.127543

[emi470079-bib-0063] Ye, W. , J. Tan , X. Liu , et al. 2011. “Temporal Variability of Cyanobacterial Populations in the Water and Sediment Samples of Lake Taihu as Determined by DGGE and Real‐Time PCR.” Harmful Algae 10, no. 5: 472–479.

[emi470079-bib-0064] Yin, W. , Y. Wang , L. Liu , and J. He . 2019. “Biofilms: The Microbial “Protective Clothing” in Extreme Environments.” International Journal of Molecular Sciences 20, no. 14: 3423. 31336824 10.3390/ijms20143423 PMC6679078

[emi470079-bib-0065] Zepernick, B. N. , E. R. Gann , R. M. Martin , et al. 2021. “Elevated pH Conditions Associated With Microcystis Spp. Blooms Decrease Viability of the Cultured Diatom *Fragilaria crotonensis* and Natural Diatoms in Lake Erie.” Frontiers in Microbiology 12: 598736. 33717001 10.3389/fmicb.2021.598736 PMC7943883

