# Supplementary material for: Temporal and Depth‐Driven Variability of Pelagic Bacterial Communities in Lake Erie: Biofilm and Plankton Dynamics
Source: Environ Microbiol Rep. 2025 Mar 21;17(2):e70079. doi: 10.1111/1758-2229.70079 (PMC11926571; doi:10.1111/1758-2229.70079)
Supplement: Supplementary file 1 — Data S1 Supporting Information. [file EMI4-17-e70079-s001.docx]

**Supplementary Information**


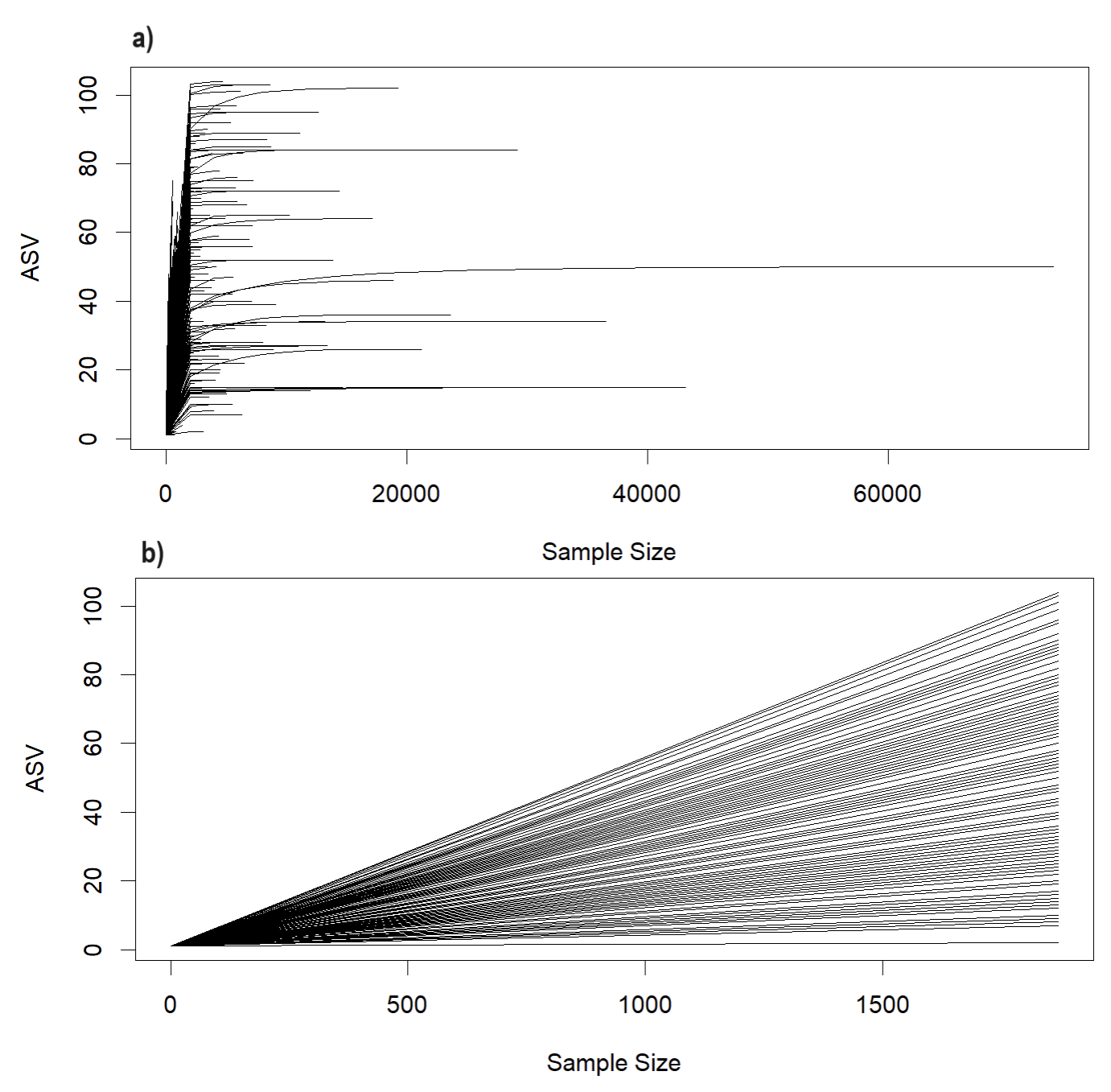


Supplementary Information 1. ASV sample size of all samples before (a – top) and after (b – bottom) rarefaction.

Supplementary Information 2. Summary of taxonomic richness indices (Choa1) and alpha diversity (Shannon) estimates corresponding to individual samples.

| Type | Depth (m) | Sampling Event | Observed | Chao1 | se.chao1 | Shannon |
| --- | --- | --- | --- | --- | --- | --- |
| Biofilm | 1 | 1 | 123 | 123.55 | 0.99 | 4.23 |
| Biofilm | 1 | 2 | 219 | 245.76 | 10.52 | 4.58 |
| Biofilm | 1 | 3 | 265 | 342.52 | 21.23 | 3.93 |
| Biofilm | 1 | 4 | 342 | 441.17 | 23.76 | 4.87 |
| Biofilm | 1 | 5 | 125 | 126.00 | 1.58 | 4.03 |
| Biofilm | 1 | 7 | 304 | 347.56 | 13.33 | 4.81 |
| Biofilm | 1 | 8 | 244 | 303.62 | 17.48 | 3.61 |
| Biofilm | 1 | 9 | 246 | 304.72 | 17.92 | 3.71 |
| Biofilm | 1 | 10 | 367 | 414.12 | 13.14 | 5.26 |
| Biofilm | 1 | 11 | 310 | 369.08 | 16.90 | 4.49 |
| Biofilm | 1 | 12 | 369 | 445.52 | 19.95 | 5.38 |
| Biofilm | 1 | 13 | 288 | 375.85 | 23.50 | 4.70 |
| Biofilm | 1 | 14 | 384 | 443.41 | 15.77 | 5.40 |
| Biofilm | 2 | 1 | 136 | 140.59 | 3.57 | 4.11 |
| Biofilm | 2 | 2 | 225 | 250.00 | 9.33 | 4.46 |
| Biofilm | 2 | 3 | 184 | 193.75 | 5.19 | 3.89 |
| Biofilm | 2 | 5 | 298 | 342.53 | 13.45 | 4.82 |
| Biofilm | 2 | 6 | 166 | 170.38 | 3.26 | 4.33 |
| Biofilm | 2 | 7 | 307 | 373.64 | 17.91 | 4.65 |
| Biofilm | 2 | 8 | 235 | 257.02 | 8.67 | 4.44 |
| Biofilm | 2 | 9 | 305 | 350.50 | 13.56 | 5.01 |
| Biofilm | 2 | 10 | 214 | 228.51 | 6.66 | 4.54 |
| Biofilm | 2 | 11 | 372 | 507.21 | 30.07 | 4.48 |
| Biofilm | 2 | 12 | 280 | 343.33 | 16.95 | 3.29 |
| Biofilm | 2 | 13 | 222 | 229.60 | 4.69 | 4.90 |
| Biofilm | 2 | 14 | 406 | 490.67 | 19.60 | 5.36 |
| Biofilm | 3 | 1 | 169 | 193.38 | 10.16 | 3.85 |
| Biofilm | 3 | 2 | 250 | 317.86 | 20.50 | 4.50 |
| Biofilm | 3 | 3 | 147 | 148.50 | 1.65 | 3.85 |
| Biofilm | 3 | 5 | 312 | 378.44 | 18.15 | 4.94 |
| Biofilm | 3 | 6 | 280 | 322.06 | 13.60 | 4.76 |
| Biofilm | 3 | 7 | 331 | 399.25 | 18.75 | 5.07 |
| Biofilm | 3 | 8 | 385 | 508.76 | 28.18 | 5.16 |
| Biofilm | 3 | 9 | 278 | 313.10 | 11.36 | 4.84 |
| Biofilm | 3 | 10 | 333 | 375.05 | 12.81 | 5.26 |
| Biofilm | 3 | 11 | 289 | 313.61 | 9.04 | 5.14 |
| Biofilm | 3 | 12 | 219 | 229.97 | 5.76 | 4.80 |
| Biofilm | 3 | 13 | 332 | 400.69 | 18.47 | 4.82 |
| Biofilm | 3 | 14 | 203 | 206.18 | 2.52 | 4.67 |
| Biofilm | 4 | 1 | 138 | 151.50 | 6.95 | 3.53 |
| Biofilm | 4 | 2 | 200 | 245.00 | 15.95 | 4.25 |
| Biofilm | 4 | 3 | 167 | 173.65 | 4.35 | 4.19 |
| Biofilm | 4 | 5 | 250 | 271.50 | 8.73 | 4.90 |
| Biofilm | 4 | 7 | 302 | 407.59 | 26.08 | 4.61 |
| Biofilm | 4 | 8 | 410 | 525.83 | 25.69 | 5.41 |
| Biofilm | 4 | 9 | 346 | 428.19 | 20.82 | 5.12 |
| Biofilm | 4 | 10 | 331 | 408.29 | 20.70 | 5.05 |
| Biofilm | 4 | 11 | 391 | 523.31 | 28.83 | 4.96 |
| Biofilm | 4 | 12 | 244 | 300.27 | 16.81 | 4.17 |
| Biofilm | 4 | 13 | 360 | 426.64 | 17.91 | 5.34 |
| Biofilm | 4 | 14 | 283 | 313.62 | 11.10 | 5.15 |
| Biofilm | 5 | 1 | 95 | 96.33 | 1.56 | 2.99 |
| Biofilm | 5 | 2 | 240 | 273.73 | 11.68 | 4.58 |
| Biofilm | 5 | 3 | 216 | 223.47 | 4.15 | 4.49 |
| Biofilm | 5 | 4 | 442 | 720.10 | 53.50 | 5.29 |
| Biofilm | 5 | 5 | 273 | 291.43 | 7.59 | 4.99 |
| Biofilm | 5 | 6 | 181 | 181.21 | 0.53 | 4.69 |
| Biofilm | 5 | 7 | 146 | 177.54 | 12.95 | 3.49 |
| Biofilm | 5 | 8 | 225 | 287.31 | 18.16 | 3.89 |
| Biofilm | 5 | 9 | 447 | 549.56 | 22.45 | 5.60 |
| Biofilm | 5 | 10 | 399 | 571.07 | 36.35 | 5.14 |
| Biofilm | 5 | 11 | 191 | 219.67 | 11.37 | 4.23 |
| Biofilm | 5 | 12 | 188 | 195.67 | 4.41 | 4.23 |
| Biofilm | 5 | 13 | 131 | 131.88 | 1.25 | 4.29 |
| Biofilm | 5 | 14 | 149 | 149.15 | 0.44 | 4.49 |
| Biofilm | 6 | 1 | 133 | 145.04 | 6.36 | 3.19 |
| Biofilm | 6 | 2 | 220 | 245.64 | 9.81 | 4.75 |
| Biofilm | 6 | 3 | 280 | 305.17 | 9.09 | 4.91 |
| Biofilm | 6 | 4 | 285 | 355.60 | 19.41 | 4.74 |
| Biofilm | 6 | 5 | 123 | 130.43 | 4.69 | 3.09 |
| Biofilm | 6 | 6 | 366 | 467.39 | 23.99 | 5.18 |
| Biofilm | 6 | 7 | 318 | 391.37 | 18.85 | 4.91 |
| Biofilm | 6 | 8 | 237 | 334.13 | 26.61 | 4.04 |
| Biofilm | 6 | 9 | 287 | 402.56 | 29.61 | 4.49 |
| Biofilm | 6 | 11 | 256 | 351.02 | 25.99 | 4.28 |
| Biofilm | 6 | 12 | 135 | 159.79 | 11.03 | 2.24 |
| Biofilm | 6 | 13 | 83 | 83.00 | 0.00 | 3.22 |
| Biofilm | 6 | 14 | 310 | 405.39 | 24.92 | 4.61 |
| Biofilm | 7 | 1 | 103 | 110.50 | 5.08 | 3.28 |
| Biofilm | 7 | 2 | 148 | 152.88 | 3.77 | 4.45 |
| Biofilm | 7 | 3 | 176 | 186.00 | 5.47 | 4.31 |
| Biofilm | 7 | 4 | 296 | 376.16 | 20.92 | 4.23 |
| Biofilm | 7 | 5 | 141 | 207.96 | 24.07 | 2.33 |
| Biofilm | 7 | 6 | 186 | 188.87 | 2.49 | 4.67 |
| Biofilm | 7 | 7 | 211 | 253.98 | 13.69 | 3.85 |
| Biofilm | 7 | 8 | 234 | 352.70 | 30.99 | 3.87 |
| Biofilm | 7 | 10 | 107 | 114.58 | 5.45 | 3.47 |
| Biofilm | 7 | 11 | 312 | 412.26 | 24.94 | 4.62 |
| Biofilm | 7 | 12 | 214 | 326.59 | 32.51 | 3.83 |
| Biofilm | 7 | 13 | 270 | 350.72 | 22.01 | 4.28 |
| Biofilm | 7 | 14 | 247 | 310.61 | 19.04 | 4.45 |
| Biofilm | 8 | 1 | 160 | 164.79 | 3.61 | 4.17 |
| Biofilm | 8 | 2 | 199 | 209.16 | 5.46 | 4.52 |
| Biofilm | 8 | 3 | 255 | 287.60 | 10.97 | 4.62 |
| Biofilm | 8 | 4 | 162 | 178.71 | 8.51 | 4.14 |
| Biofilm | 8 | 6 | 270 | 325.35 | 16.03 | 4.08 |
| Biofilm | 8 | 7 | 182 | 307.14 | 41.29 | 3.83 |
| Biofilm | 8 | 8 | 256 | 422.05 | 41.46 | 4.09 |
| Biofilm | 8 | 10 | 91 | 91.00 | 0.10 | 3.68 |
| Biofilm | 8 | 11 | 406 | 481.46 | 18.93 | 5.54 |
| Biofilm | 8 | 12 | 256 | 353.00 | 25.94 | 3.87 |
| Biofilm | 8 | 13 | 303 | 422.10 | 28.38 | 4.32 |
| Biofilm | 8 | 14 | 240 | 298.90 | 18.51 | 4.34 |
| Plankton | 1 | 2 | 185 | 221.83 | 13.41 | 4.30 |
| Plankton | 1 | 5 | 263 | 384.88 | 30.70 | 4.17 |
| Plankton | 1 | 6 | 198 | 273.14 | 26.76 | 4.12 |
| Plankton | 1 | 9 | 163 | 176.00 | 6.71 | 4.06 |
| Plankton | 1 | 11 | 329 | 495.85 | 37.59 | 4.53 |
| Plankton | 1 | 12 | 183 | 217.03 | 12.64 | 3.81 |
| Plankton | 8 | 2 | 158 | 182.79 | 11.03 | 4.10 |
| Plankton | 8 | 4 | 220 | 283.90 | 19.84 | 4.13 |
| Plankton | 8 | 5 | 218 | 274.69 | 18.21 | 4.27 |
| Plankton | 8 | 6 | 144 | 149.06 | 3.78 | 4.07 |
| Plankton | 8 | 9 | 207 | 239.69 | 12.04 | 4.27 |
| Plankton | 8 | 13 | 269 | 354.56 | 23.39 | 4.50 |


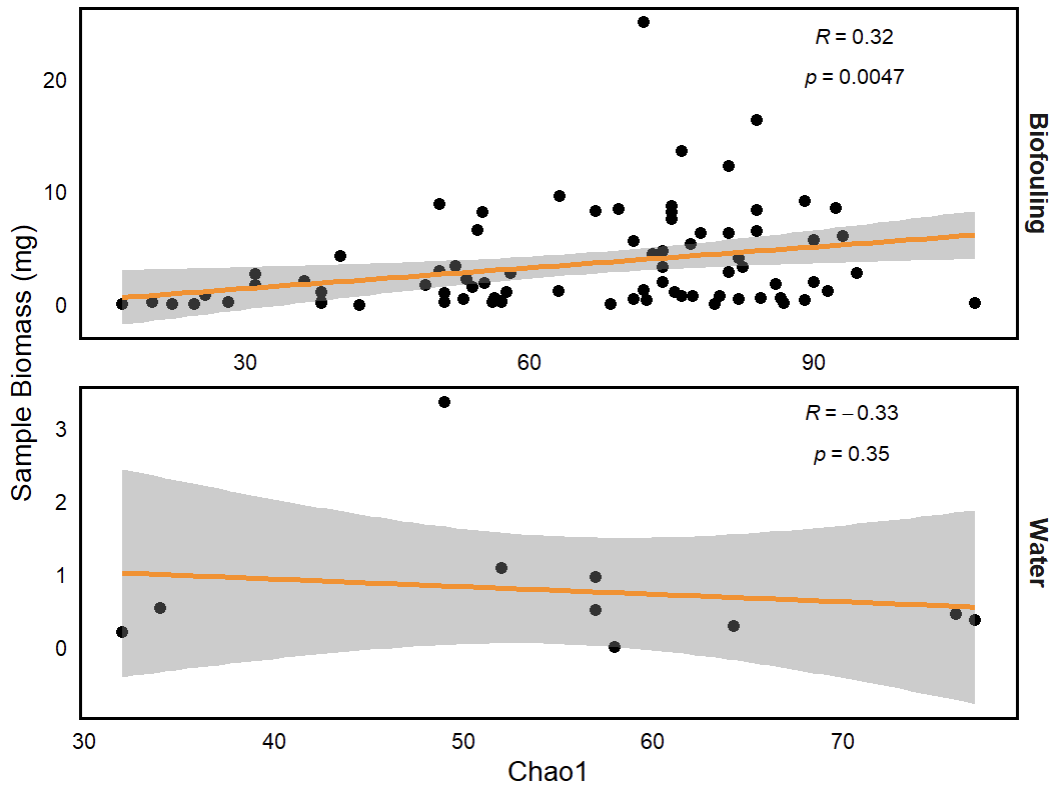


Supplementary Information 3. Spearman’s correlation between sample biomass (in mg) and calculated Chao1 richness from rarefied data for biofilm (biofouling – top) and plankton (water – bottom) samples.


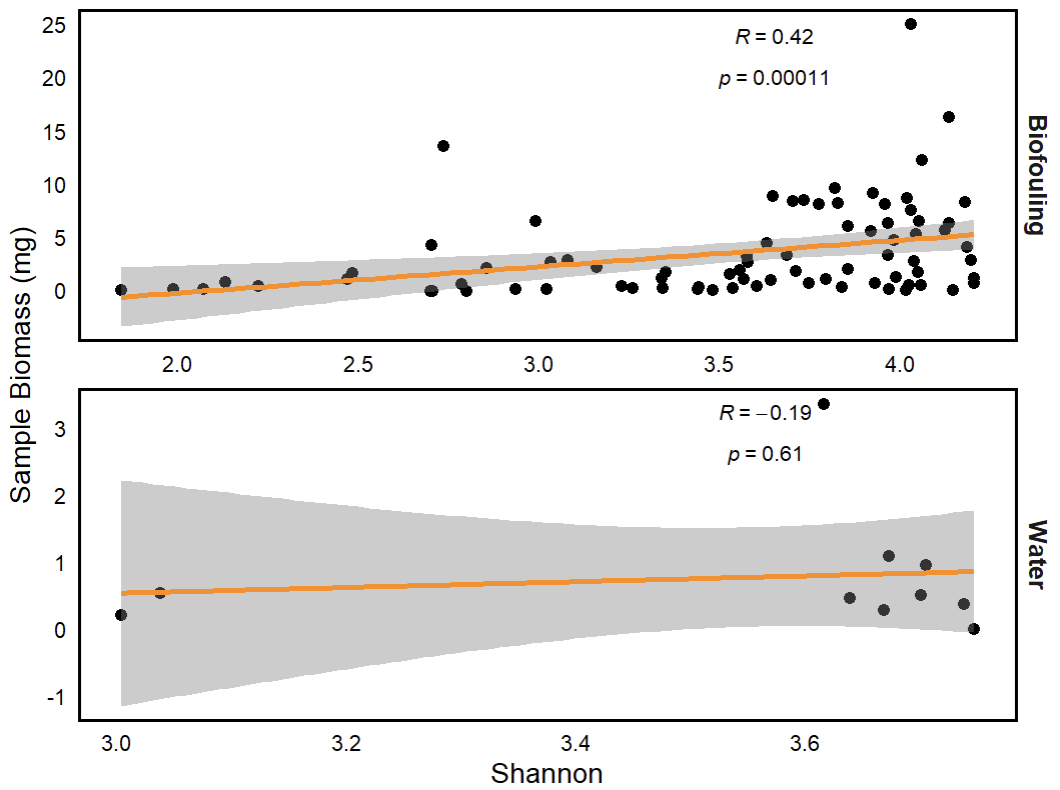


Supplementary Information 4. Spearman’s correlation between sample biomass (in mg) and calculated Shannon Index from rarefied data for biofilm (biofouling – top) and plankton (water – bottom) samples.

**
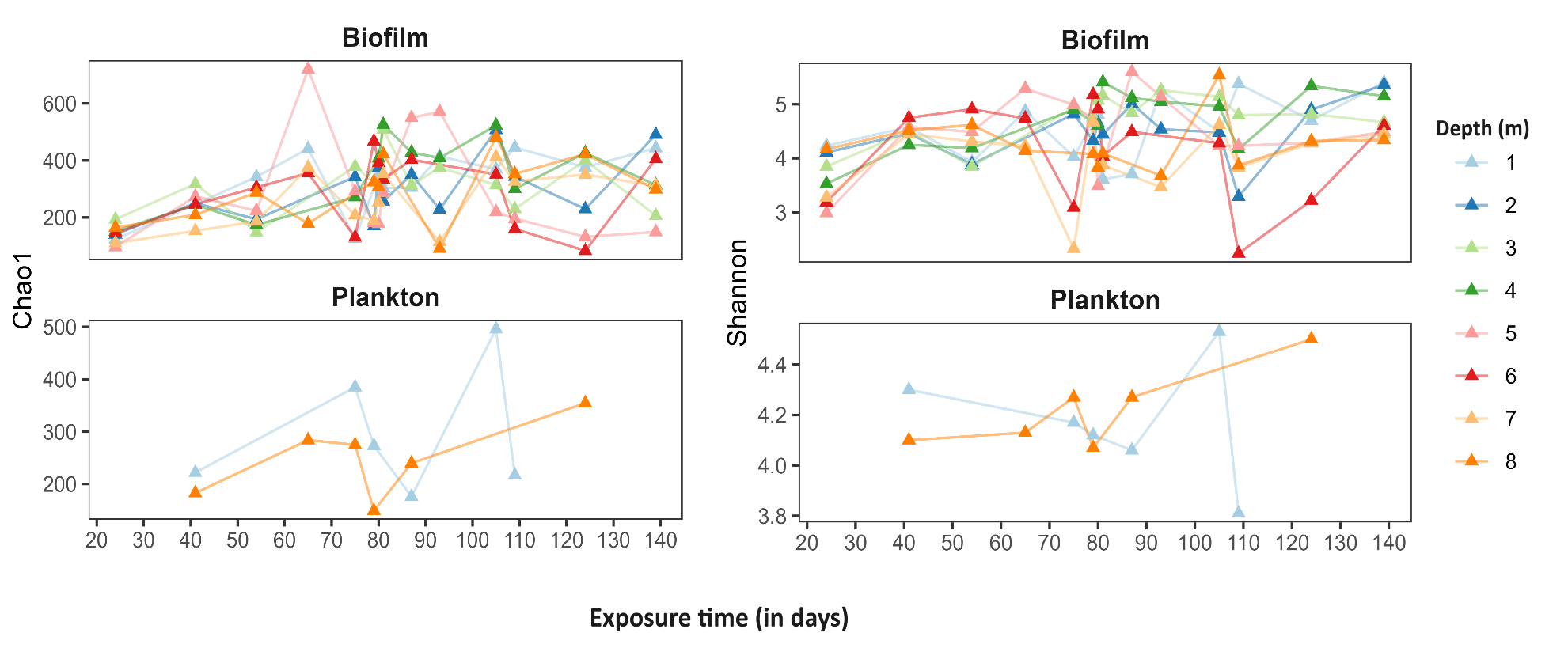
**

Supplementary Information 5. Changes in Chao1 diversity (top left, bottom left) and Shannon’s diversity (top right, bottom right) over the study duration. Start of exposure time begins when substratum was deployed (22 May 2022).


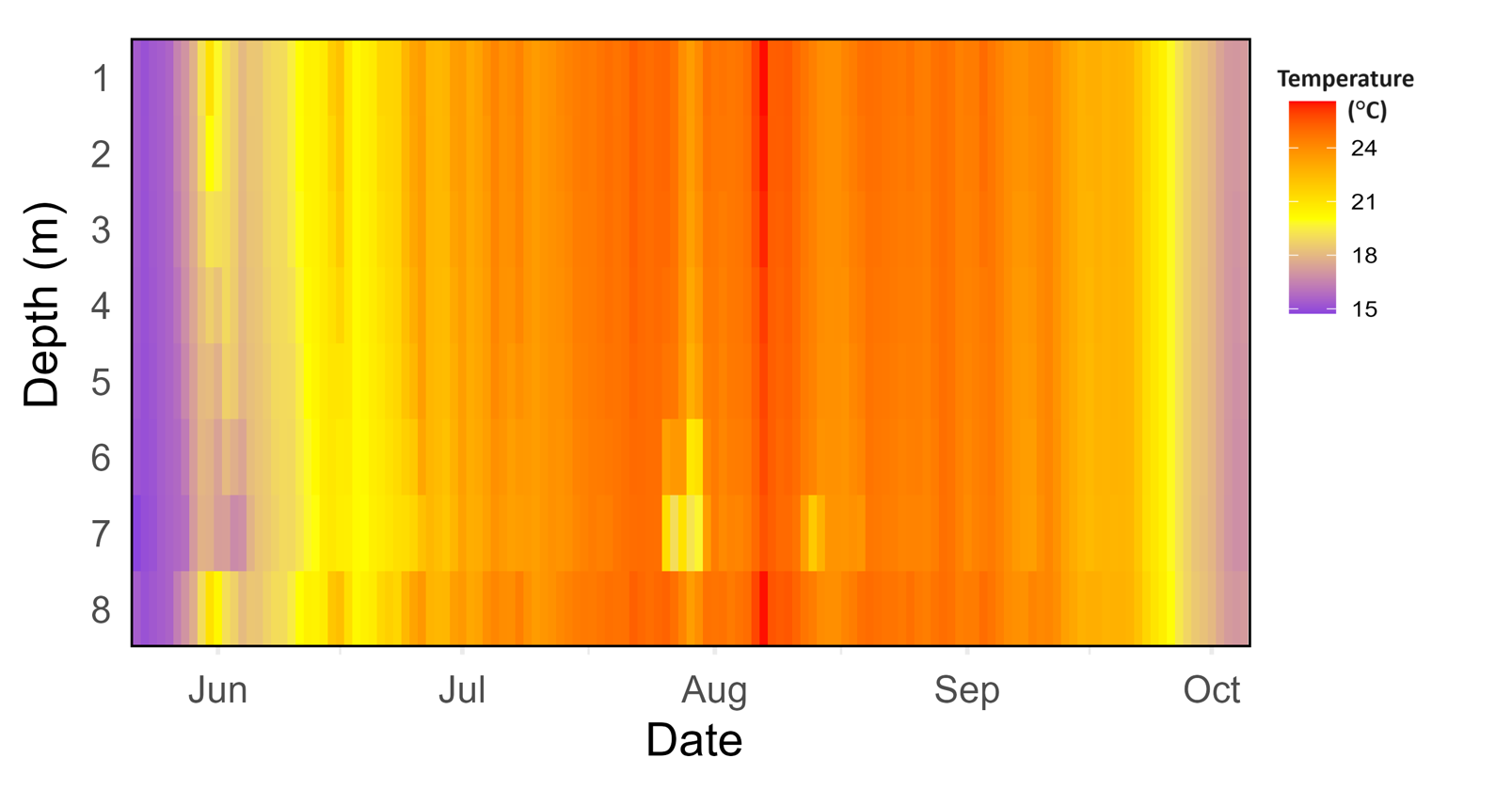


Supplementary Information 6. Daily temperature profile at Site 6. Temperature loggers were positioned at 1m increments from 1m below surface to 1m above bottom. RBR solo^3^ loggers (±0.002°C accuracy) were used from depths 1 – 7m, while Innovasea aquaMeasure DO sensor (±0.2°C accuracy) was used at the 8m depth. Temporal profile indicates that the site location is relatively polymictic with short-term (3-5 day) stratification around 7m in lake July.
